# How HIV Clients Find Their Way Back to the ART Clinic: A Qualitative Study of Disengagement and Re-engagement with HIV Care in Malawi

**DOI:** 10.1007/s10461-021-03427-1

**Published:** 2021-08-17

**Authors:** Stephanie Chamberlin, Misheck Mphande, Khumbo Phiri, Pericles Kalande, Kathryn Dovel

**Affiliations:** 1 Department of Health and Behavioral Sciences, University of Colorado Denver, Campus Box 188, P.O. Box 173364, Denver, CO 80217-3364 USA; 2 Partners in Hope Medical Center, Lilongwe, Malawi; 3 Department of Medicine and Division of Infectious Diseases, David Geffen School of Medicine at University of California Los Angeles, Los Angeles, CA USA

**Keywords:** Re-engagement, Retention, Return to care, Sub-Saharan Africa, HIV care

## Abstract

Retention in antiretroviral therapy (ART) services is critical to achieving positive health outcomes for individuals living with HIV, but accumulating evidence indicates that individuals are likely to miss ART appointments over time. Thus, it is important to understand why individuals miss appointments and how they re-engage in HIV care. We used in-depth interviews with 44 ART clients in Malawi who recently missed an ART appointment (> 14 days) but eventually re-engaged in care (within 60 days) to explore reasons for missed appointments and barriers and facilitators to re-engagement. We found that most individuals missed ART appointments due to unexpected life events such as funerals, work, and illness for both clients and their treatment guardians who were also unable to attend facilities. Several reasons differed by gender—work-related travel was common for men, while caring for sick family members was common for women. Barriers to re-engagement included continued travel, illness, and restricted clinic schedules and/or staff shortages that led to repeat facility visits before being able to re-engage in care. Strong internal motivation combined with social support and reminders from community health workers facilitated re-engagement in HIV care.

## Introduction

Antiretroviral therapy (ART) initiation has increased dramatically in sub-Saharan Africa since the introduction of Universal Treatment policies in 2016, with ~ 83% of those diagnosed with HIV in the region on treatment in 2019 [1]—a remarkable success. For this success to translate into long-term viral suppression, consistent retention in HIV care is essential. Yet successfully engaging in HIV care over time is complicated—involving frequent visits to the health facility and daily ART adherence amidst other life priorities and familial demands [2, 3]. These challenges are reflected in high rates of default from ART programs in sub-Saharan Africa over time [4, 5]. One regional study estimated that only 81% of ART clients were retained in care within one year after ART initiation, and this dropped to 65% just three years after initiation [6]. Yet for many, leaving HIV care is not a fixed state [7, 8]. A recent study from the region shows that 73% of those who missed an HIV care appointment returned to care, most within one year, suggesting that cycles of disengagement and re-engagement may be central to the ART experience [9].

While high rates of re-engagement are promising, even short periods of absence from care remain concerning. People living with HIV (PLWH) who miss ART appointments are more likely to have poor ART adherence leading to increased risk for ART resistance and viral transmission in the short-term, and increased risk of remaining out of care and HIV-related morbidity and mortality in the long-term [2, 9–11].

Public health efforts to encourage PLWH to return to HIV care as soon as possible require a deeper understanding of barriers and facilitators to re-engagement. Well established barriers to retention in HIV care (but not to re-engagement) include fear of unwanted disclosure and HIV-related stigma, long wait times at health facilities, distance to facilities and lack of transportation, poor quality of HIV services provided, treatment fatigue and side effects, and familial or social responsibilities that inhibit facility attendance [12–14]. Conversely, facilitators to retention and adherence include tangible social support in the form of money or transportation to the facility, and family or friends who express their expectations/desires that the person living with HIV should prioritize their own health [15, 16]. Yet the specific barriers and facilitators to re-engagement for PLWH who have already missed ART appointments are less clear [17, 18]. The limited available research shows that HIV clients struggle to re-engage in care due to shame and fear of criticism from health care providers [2, 19] and treatment fatigue [18]. Among the few studies in the region that specifically explore how PLWH successfully re-engage with care, there is evidence that kind health facility staff, social support, outreach from health facilities, and more flexible options for clients to refill ART prescriptions are important facilitators of re-engagement [20–23]. Given the small and location-specific nature of these studies, it remains unclear if these findings will translate to other contexts in the region. In this study, we examine the challenges and successes of PLWH who effectively re-engage with HIV care after missing routine ART appointments in Malawi.

Factors contributing to disengagement from, and re-engagement in, HIV care may differ by gender. First, health facilities tend to be structured around women’s and children’s health care needs, creating an environment that is more familiar and comfortable for women [24, 25]. Second, familial and work responsibilities are often divided by gender, suggesting that men and women have differing priorities to balance alongside HIV care. Women are primarily caregivers for children and other family members, including during times of illness [26, 27]. Men, on the other hand, are more often the primary ‘bread winner’, requiring more time working and travelling away from home [28]. Gendered health facilities and social responsibilities may result in men having less time to access HIV care, and less experience and more discomfort navigating facility-based barriers to care [24, 29]. Similarly, women’s unpaid care work may require them to miss ART appointments in order to care for family members [26, 28]. However, women may be more apt to re-engage in services since they often attend health facilities for other services already [25] and may be more comfortable communicating with health facility staff during re-engagement.

In this study, we use in-depth interviews with PLWH to understand the dynamic cycle of HIV care disengagement and re-engagement among men and women in Malawi. Amidst calls for more client-centered approaches to HIV care and treatment [30, 31], this study focuses on the client’s experiences, rationales, efforts, and resources as part of their HIV care engagement cycle. Throughout our analysis, we focus on gendered differences in PLWHs’ experiences of disengagement and re-engagement.

## Study Setting

Malawi has a HIV prevalence at 9% in the adult population [1]. In many ways, rural Malawi reflects many of the socio-economic and healthcare realities found across rural southern and eastern Africa. The HIV epidemic is situated within a context of high poverty, with 52% living below the national poverty line, high levels of fertility (an average of 4.2 pregnancies per woman), limited employment opportunities, and high rates of morbidity and mortality, with a life expectancy of 63.8 in 2018 [32]. The majority of HIV care in Malawi is decentralized and provided in rural areas [33]. The health care system is often overburdened and limited human resources are a primary concern [34].

HIV treatment is often siloed from other health services and typically only scheduled on one or two days during a week (not every day). At ART initiation, clients are strongly encouraged to identify someone who will know their HIV status and will be able to collect ART refills when the client is unable to attend (i.e., ‘treatment guardian’). If a treatment guardian attends the ART clinic on the client’s behalf, this would not be considered a missed appointment.

This study was carried out in eight rural health facilities in central (four in the Dowa/Kasungu districts) and southern (four in Chikwawa district) Malawi. Participating facilities received support delivering HIV testing and care services from Partners in Hope, a non-profit organization in Malawi. At the time of the study, each facility had a designated ART clinic to routinely distribute ART and monitor HIV clients’ health. The two larger health facilities provided routine ART services three and five days per week, while the six smaller health facilities only offered routine ART services one or two days per week.

## Methods

### Study Design

This qualitative research was nested within a larger mixed-methods study that assessed patterns of HIV care and their correlates among new ART clients in Malawi. For this sub-study, in-depth interviews were conducted with men and non-pregnant/non-breastfeeding women who initiated ART, had been at least two weeks late for a routine ART appointment, and re-engaged in HIV care prior to study recruitment. Two weeks is a standard timeframe within Malawi for flagging late appointments based on Ministry of Health guidelines [35, 36]. We focus on non-pregnant/non-breastfeeding women because ART services have been integrated into antenatal care for nearly a decade, resulting in different barriers and facilitators to re-engagement for these women.

### Interview Sampling Strategy

We used purposive sampling to select potential respondents and stratified our sample by gender (men/women) and region (central/southern) to ensure both genders and regions of Malawi were represented. At the time of study recruitment, eligible clients: (1) were ≥ 15 years of age; (2) had initiated ART for the first time in the last 12-months (under the Universal Treatment policy implemented in Malawi in July of 2016); (3) were  >14 days late for an ART appointment in the same 12-month period; (4) had returned to HIV care within 60 days after a late ART appointment; and (5) were non-pregnant/non-breastfeeding. The research team reviewed routine medical records to identify and recruit potentially eligible PLWH at participating ART clinics.

### Interview Data Collection

Interviews took place in private rooms at the health facility. All interviews were conducted by local research assistants in the local language (Chichewa). Interviewers and respondents were matched by gender. The interviews were audio recorded, with the permission of the respondent. Oral consent was ascertained from all respondents, and respondents were informed of their right to refuse participation in any part of the study at any time. Participants were given refreshments during the interview, but did not receive any other incentive or compensation. As all interviews took place at the clinic, there were peer counselors or other clinic staff available for referrals to additional services and counseling as needed.

We developed an in-depth interview guide by examining existing literature and gathering input from community-based stakeholders, including health facility staff. We took a narrative approach to in-depth interviews [37], asking individuals to share their story, to provide contextual details about what was going on in their lives at the time of the missed appointment, and how they returned to their primary ART clinic. We adapted the guide iteratively throughout the interview process as needed. In this paper, we explore the following domains from the interview guide: (1) reasons for missed ART appointment(s); (2) barriers to re-engagement; (3) what facilitated their return to care; (4) any positive or negative experiences with providers upon returning to the clinic; and (5) perceived risks and benefits regarding ART adherence, including concerns regarding missed ART doses.

### Data Analysis

Interviews were simultaneously transcribed and translated into English. Quality checks were conducted as close to the time of data collection as possible (within two weeks) in order to identify transcription or translation errors, provide feedback to research assistants, and clarify any confusing information from the interview.

After data collection was completed, two study team members read each transcript and created summary memos to understand respondents’ narratives about their missed appointments and re-engagement in care. After completing summary memos, we developed an analytic codebook based on a-priori themes covered in the interview guide (deductive) and emergent themes identified during the creation and review of summary memos (inductive). We systematically coded transcripts using Atlas.ti 8 in two phases: First, we applied overarching codes to broadly categorize text related to the main domains in the interview guide. Second, within each overarching code, two study team members applied detailed analytic codes from the developed codebook. We randomly selected 15/44 of interviews to double code and review for consistency and quality. The study team discussed discrepancies and applied any adjustments in a final round of coding.

### Ethics

Ethical approval was attained by the Institutional Review Board at University of California Los Angeles (UCLA) and the National Health Science Review Committee (NHSRC) in Malawi.

## Results

21 men and 23 women completed in-depth interviews between July and September of 2017 (44 interviews in total). Respondents included a range of socio-economic and demographic characteristics (see Table 1). The majority of respondents were at or below 40 years of age, were in an on-going relationship, and had more than one child. More men than women reported being in an on-going relationship, and the majority of their partners were also taking ART. Most respondents had a primary level education, and men reported more years of schooling when compared to women. Finally, the vast majority of respondents reported travelling an hour or more to reach their ART clinic.

**Table 1:** Demographic characteristics

	Women	Men	Total
Age			
18–30	8	8	16
31–40	8	6	14
41+	7	7	14
On-going relationship	13	16	29
Current partner HIV+ and On ART	7	14	21
Number of children			
0–2	6	9	15
3–6	13	10	23
> 6	4	2	6
Education level			
Low (0–3 years)	14	7	21
Medium (4–7 years)	8	12	20
High (8+ years)	1	2	3
Distance to clinic: > 1 hour	17	15	32
**Total**	**23**	**21**	**44**

Below we describe reasons for missed appointments and barriers and facilitators to re-engaging in HIV care. Respondent narratives about missed ART appointments highlighted the complex decisions PLWH make when balancing HIV care and other personal and familial demands. While we present findings as separate themes, it is important to recognize that these were not neatly segmented barriers and facilitators, but rather inter-related events and dynamic processes of disengagement and re-engagement, as demonstrated in the following summary:

A 28-year-old single mother of five children, Josephine [pseudonym], earns money by doing odd-jobs and chores for others. She was diagnosed with HIV in 2015 and began taking ART in 2016. She makes every effort to attend her monthly ART appointments, including asking a friend to go to the clinic in her stead when she was recently too sick to go herself. In the past year, she missed an ART appointment because of travel to attend a funeral. At that time, her friend was also attending a different funeral and Josephine’s official treatment guardian (a family member) was attending the same funeral with Josephine. So, she was not able to find anyone to go to the clinic for her on her appointment date, and she believed that she was not allowed to go to the clinic before her scheduled appointment. She was somewhat concerned that she would be ‘shouted at’ by the clinic staff for missing her appointment. Nonetheless, she was motivated to return to the clinic as soon as possible after returning home from the funeral because she was concerned that she might get sick if she did not continue with her ART. (28-year-old, unmarried woman).

**Fig. 1:**
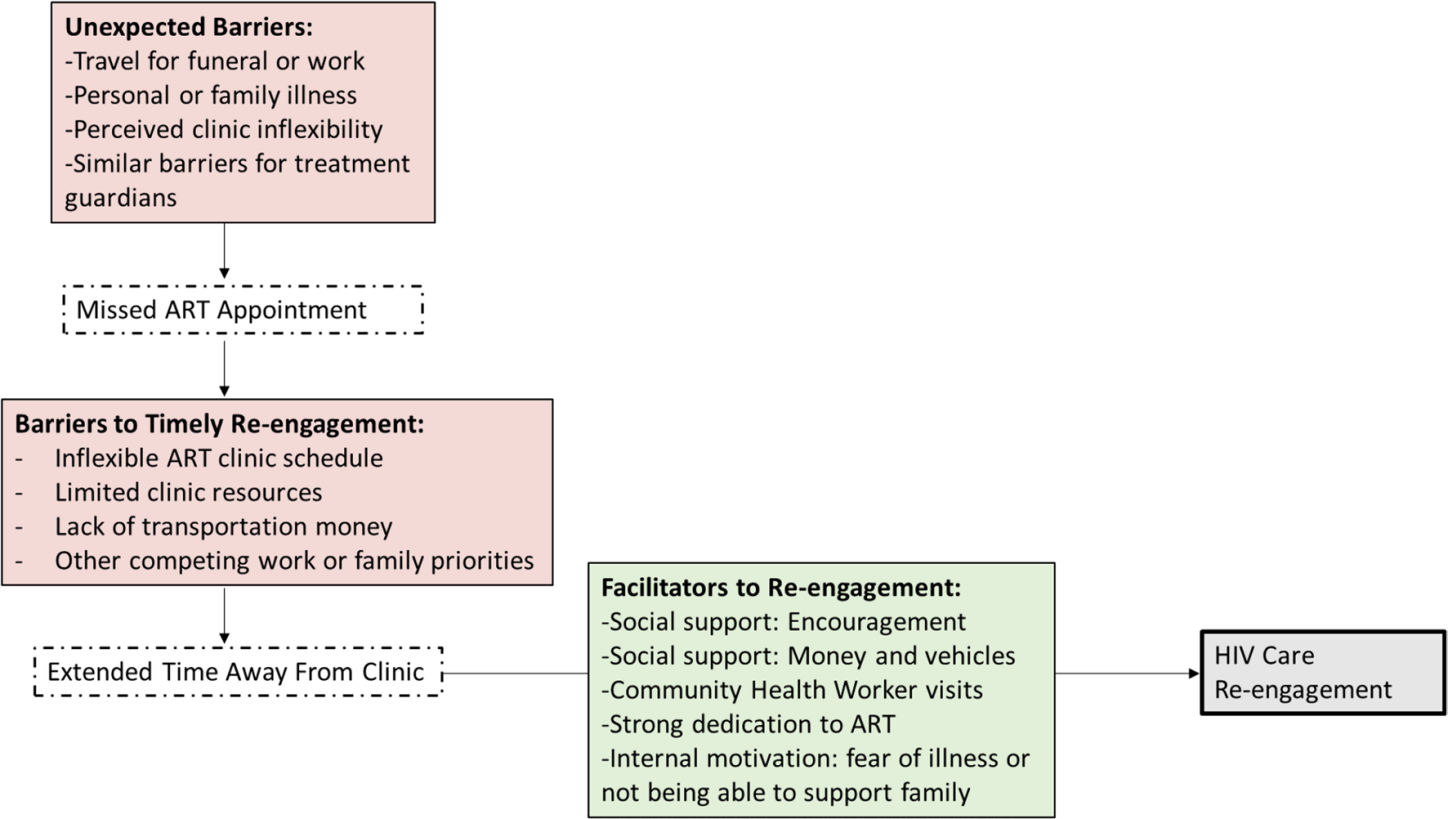
Summary of barriers to ART appointments and facilitators for re-engagement

Figure 1 summarizes the key findings presented below.

### Perceived Importance of ART and Regret About Missed ART Appointments

Nearly all participants stated that adherence to ART was a high priority. With few exceptions, respondents believed that treatment adherence was critical to their health and the well-being of their families, and desired to adhere to medication even when faced with competing life priorities. Respondents generally expressed anxiety, disappointment, and regret when reflecting on their missed ART appointments and the risk of missing ART doses.

“Because ARVs are our life. When we are taking ARVs we become healthy." (34-year-old, unmarried woman)

“It just happened that I missed that time, but I was very disappointed to miss the date. I don’t want to miss, I want to be taking the medicine every day." (34-year-old, married man)

“I really wanted to go collect the drugs. I knew that even if I go to do piece work it won’t work because I will still think about the collection of the drugs because the drugs are my life." (32-year-old, married woman)

### Reasons for Missing Routine ART Appointments

Reasons for missed appointments were often multi-faceted, involving overlapping barriers. Most respondents expressed that they did not intend to stop or pause their HIV treatment, but that other life demands and circumstances made it difficult or impossible to attend their ART appointments on-time. When asked why they missed an ART appointment, respondents cited needing to provide for their family, caring for sick relatives, attending funerals, and managing other (non-HIV) health issues.

#### Unexpected Travel and Obligations

For just under half of respondents, unexpected travel away from home created a direct barrier to attending ART appointments. Reason for travel varied by gender. Both men and women reported travelling to attend funerals during the time of their ART appointment, while a third of men and no women reported work-related travel as their main reason for missing their ART appointment.

“When I [returned] here the doctor said that, ‘we told you to come and get drugs but up to two months has passed’. I told him that I am sorry doctor—one of the months I was at a funeral, the other one I was just reluctant, please forgive me." (58-year-old, unmarried woman)

"Okay, what happened is that...I work as a builder... So that I can support my family. I find [piece work] in Mozambique. So I was worried about waiting for the payments and when they paid us, I went back home, and it happened that I missed my appointment dates here." (46-year-old, married man)

Most respondents indicated that they were unaware that they could seek an emergency ART refill at another clinic while traveling, an option available to clients as part of Malawi’s national ART program. Some reported that they believed they could not attend another clinic without their health passport, which was often forgotten or left at home.

#### Illness

Respondents’ own health concerns, usually outside of HIV, also posed a barrier to attending ART appointments. A handful of both men and women reported not being able to attend their ART appointments due to both minor and major personal illnesses.

“What made me fail to come here to collect drugs is that the leg was swollen." (47 year-old, unmarried woman)

“I was sick that month. When I got sick, I saw that there was no person who I could ask to receive the drugs for me. It was just a sickness, but it wasn’t a serious problem." (50-year-old, unmarried man)

On the other hand, with one exception, only women reported missing an appointment because they were caring for a sick family member. Over a quarter of women reported a variety of ways that caring for the health of family members interfered with attending their routine ART appointments.

“I was with my in-law at the hospital in [nearby town]. She was pregnant." (52-year-old, unmarried woman)

“My child has a mental disorder…I decided to go to [a religious healing center in another location]—maybe they could help…But the [religious healing center] doesn’t allow someone to take drugs. Even acetaminophen, [they say] ‘don’t stay here, go!’ " (59-year-old, unmarried woman)

#### Barriers were Similar for Treatment Guardians

Treatment guardians are meant to offer an important fall back for ART clients when they are unable to attend the ART clinic. However, we found that within close social networks, treatment guardians were often impacted by the same barriers experienced by the respondents; meaning that treatment guardians were also unavailable to help access needed treatment. For example, treatment guardians may have attended the same funeral service, or experienced an infectious illness that overlapped with the respondent’s illness. This pattern held for one-third of women, and just under a quarter of men.

“My husband got very sick…I also got sick with flu so I was weak. So it was difficult for me to go to the hospital because my partner was [also] sick. So it was difficult to find someone who could [go] to the clinic." (32-year-old, married woman)

"…even if [my wife] came to pick up medications for me while I was still [in Mozambique], she would just end up keeping them at home. It was hard because of transportation." (46-year-old, married man)

“The river had flooded and I failed to cross and come here to pick up medication…. I did think about the guardian, because we both live in the same community, so they too failed to cross the river. …. Uh, there wasn't any alternative since we only come on Wednesdays and I had to wait." (35-year-old, unmarried woman)

#### Distance to Facilities and Daily Work were not Barriers to Retention

While distance to facilities was seen as a challenge, it was manageable for the vast majority of respondents in our study and did not result in missed appointments (with the exception of two men). Instead, men and women described various strategies to procure transportation from their home to the clinic during the course of daily life, when not experiencing unexpected life events.

“I find money to use for transport when coming here. So when I see that days are approaching, maybe its next week, I search for money which I can use for coming here. When I don’t have money for coming here, I take the maize and sell it, then get money for coming here. [Or] I just go to [local village] where my friends are and I borrow money." (44-year-old, married man)

“If we have money, we board a car. If we don’t have transport, we walk…If we don’t have [money], we just walk...As long as we receive the medication, [it doesn’t matter if we] walk." (26-year-old, married woman)

Most respondents, both men and women, were able to overcome challenges related to day-to-day tasks at home (not including unexpected travel or illness)—such as routine child care, cooking, tending to the garden, or piece work—by shifting tasks to another time or to another family member. This is in contrast to the demands of external employment and unexpected/non-routine familial responsibilities, which posed the greatest barriers to maintaining routine ART appointments.

“When I am away [at the clinic], I find that [my husband or in-law] have made the child take a bath, given her food, and kept some food for me as well." (20-year-old, married woman)

“I leave the chores because I can’t do chores and also prepare to leave for the hospital; it can’t work with regards to time. I cancel all the chores and go to the hospital. Maybe there are [clients] at home so I can help them with medicine [note: he is a traditional healer]. I tell them to go back and come back the next day because there is some place I would like to go. Or maybe I had to build a bathroom, then I don’t even start and do it after I come back. Maybe I have to collect sand for my toilet; I leave that as well so that I should first go to the hospital." (34-year-old, married man)

### Barriers to Re-engagement

Pathways back to ART services were not always simple. When asked how they made it back to the clinic, numerous respondents described unexpected obstacles and repeated efforts that were required in order to return to care. These obstacles were often different from the original reasons respondents missed an appointment, and included limited clinic days and hours of operation and transportation costs to return home from extended travel. The following excerpt exemplifies how new challenges to re-engagement emerge the longer individuals were out of care:

“There, I encountered a funeral. So I spent a week there, but I had the medicine. The medicine finished while I was there. When I wanted to come and collect the medicine, I got sick. So I failed to come here because it is very far." (34-year-old, married man)

#### Limited Clinic Schedules and Resources

Respondents reported that limited clinic hours or inflexible appointment schedules constrained their ability to address their HIV care amidst unexpected life events. Nearly half of respondents, both men and women, referenced this as a broader challenge to engagement in HIV care. Below, we focus on situations where this posed a specific barrier to individuals’ re-engagement in HIV care.

Just under a quarter of respondents, equally divided between men and women, reported that they returned to the health facility as soon as possible, but were turned away because they did not come on an ART clinic day or because an ART provider was not available for various reasons. In some cases, respondents struggled to find the time or money to return to the clinic again on the next available ART clinic day.

“Since we were in mourning, we didn’t find a chance to come here. So we came in the middle of the week. They said ‘here we don’t give the ARVs in the middle of the week. You should come on Thursday. All this group of people [in the out-patient line] wants us to help. So [we cannot] stop our work to help you. You should wait for Thursday.’ So the days [between appointments] kind of increased." (47-year-old, unmarried woman)

“[When I came back] I found there was nobody [at the clinic]. Yes, it was a holiday. So, I went back after July 6th…I couldn’t get the drugs—I was met with challenges again. So, I went back twice to get the drugs." (50-year-old, unmarried man)

“For instance, when I came this month, I was told that the doctor has gone for some training and has carried the keys for the room where the ARVs are. So I went back home. So due to scarcity of transportation fare, I spent two days without coming here and then I came afterwards to pick up the medications from the hospital. [When I originally missed my appointment] I still had many pills in the bottle. So I was still taking those pills. But during the three days after returning to the clinic, when the doctor had traveled, that's when I did not take any pills." (27-year-old, married man)

#### Procuring Money for Transportation Back Home Extended Time Away from the ART Clinic

Among men who missed ART appointments due to travel for work, over half reported having difficulties procuring transportation money to return home to attend the ART clinic where they were registered.

"... because it was a long distance, I didn’t have transport money to get here and go back [to the clinic], so I just thought I should get the Irish [potato to earn money]…" (22-year-old, married man)

“I was searching for piece work [in Lilongwe], so that I can find money for the transport cost to come back here." (21-year-old, married man)

#### Negative Interactions with Clinic Staff were Concerning, but not a Barrier to Re-engagment

Numerous respondents noted concerns about clinic staff being disappointed and/or ‘shouting’ at them for missing appointments. However, no respondent cited this as a reason for delaying their return to care. Most noted the importance of simply explaining their situation to the clinic staff and receiving their ART as soon as possible.

“I was thinking that [the clinic staff] would feel like I have just stayed at home because I have not sent any message. You see, I was worried, but I [decided to] answer when they ask me. If they will not believe, then it is fine, but still I will explain why I failed to go and collect the ARVs on that day. I was worried that they would ask me questions or shout at me that I am being childish not to collect the ARVs." (21-year-old, married man)

“I didn't care whether [the clinic staff] were going to be harsh with me so long as they give me the medications. Because when you miss an appointment, they are supposed to shout at you because you missed an appointment, because they say that the medications for each week are planned for the people who have an appointment during that day." (38-year-old, married woman)

### Facilitators of Re-engagement

#### Fear of Illness and Concern for Family

When asked why they returned to HIV care, almost all respondents reported a strong internal motivation because they feared that they could become ill or die if they did not take ART. Nearly three-quarters of respondents, equally distributed between men and women, expressed a desire to remain healthy in order to provide and care for their families as their primary motivation for re-engaging with HIV care.

“I have seen the importance of taking medicine because my body becomes different if I stay without taking medicine…. my body becomes weak…But if I take the medicine, I work with a lot of energy." (40-year-old, married man)

“I thought maybe I could die anytime. That means my children will become orphans." (47 year-old, unmarried woman)

“I am caring for my life so that I should care for my family. By that I mean, if I am sick, I would not be able to care for my family." (52-year-old, married man)

“I thought that if I don’t pick up [my] medication, then something may happen to my body such that I may get sick and even die, and I may leave the children in problems." (35-year-old, unmarried woman)

#### Social Support and Encouragement to Return

Emotional and financial support from respondents’ social networks further facilitated their re-engagement. In the majority of cases, respondents disclosed their missed ART appointment to a friend or family member, and the ensuing conversations and support regarding ART services were central to their return to care. One-third of men and just under a quarter of women reported that a friend, family member, or boss encouraged them to return to the ART clinic as soon as possible**.**

“So, my relatives were explaining that I may unexpectedly get sick, unlike how a person normally gets sick. Therefore, it is better for me to go to the hospital now, while my immunity is still high and pick up medications, so that I should continue taking them. That's when I sourced some money to come here at the hospital to pick up medication." (27-year-old, married man)

“My relative said that I should go back [to the clinic] so that I should be assisted first, [and] that my life depends on the drugs." (32-year-old, married, woman)

A handful of men and women specifically noted that friends or family members offered a bike, money for transportation, or a personal escort to help them return to clinic.

“I remembered my appointment and [my neighbor] gave me a bike." (30-year-old, married woman)

“That trip, I rode a bicycle. I borrowed my boss’ bike. He knows my HIV status." (27-year-old, unmarried man)

#### Community Health Worker Outreach

Some respondents, mostly men, reported that a community health worker associated with the ART clinic encouraged them to return to care as soon as possible.

“So when the [community health worker] discovered that I am not coming, they came and picked me up to say ‘You should start taking medicine again. We are surprised that you are not coming [to the clinic].’ That is why I started taking medicine again." (40-year-old, married man)

“I came back to the clinic because when [the community health worker] visited me. I told her that I was failing to come to the clinic because my husband would not allow me to come; and she told me that on Tuesday, I should come to the clinic so that I should resume taking medication. That's when I came and resumed taking medication." (24-year-old, married woman)

## Discussion

We conducted one of the first qualitative studies to examine the experiences of men and women who disengage and successfully re-engage in HIV care in Malawi. Respondents reported initially missing ART appointments due to unexpected responsibilities that required extended travel or caregiving—life did not stop for ART. Work schedules and work-related travel were particularly relevant barriers for men, while caring for ill family members almost exclusively posed a barrier for women. Funeral attendance, and travel related to funerals, was common for both men and women. These unexpected events were often complicated by inflexible ART clinic schedules, clinic staff shortages, lack of funds for transportation when far from home, and a lack of knowledge about alternative options for ART collection (e.g., emergency ART pick-up from other health facilities). Respondents nonetheless demonstrated a dedication to continuing their ART regimens and returning to the health facility in order to remain healthy for themselves and their families. Respondents often received encouragement and transportation resources from friends and family members to help them return to care. Men, in particular, described encouragement from community health workers as a catalyst for their re-engagement in care. Findings directly address growing calls for focused research and interventions to address intermittent gaps in HIV care [7, 14, 38, 39].

The unexpected life events that kept respondents in our study from attending ART appointments are not easily postponed or curtailed to prioritize health care. In a context of economic insecurity, income-earning opportunities are essential for sustaining the health of one’s family, and care-taking responsibilities cannot simply be neglected until a later time [14]. Further, funerals in Malawi are common and often unexpected in a setting with high levels of premature mortality [40, 41]. Culturally, it is normative for funerals to take place near the time of death, and there are strong cultural expectations for all family members to attend funerals with little advanced notice [40]. While this context may be unique to life and HIV care in Malawi, the difficulties involved in managing competing life priorities alongside chronic care are a common human experience [15, 42].

Importantly, we found that treatment guardians, or individuals who support ART clients and can collect ART refills on their behalf, often experienced the same unexpected travel, illness, or caregiving constraints as the ART clients themselves. As a consequence, treatment guardians had limited ability to act as a ‘back up’ for ART clients, especially within close social networks. More research is needed to understand the role and effectiveness of treatment guardians for mitigating lapses in HIV care, and strategies to address limitations in the context of unexpected life events.

While routine tasks and distance to health facilities posed challenges to routine clinic attendance, in the context of daily living, these obstacles were not insurmountable for the men and women in our study. Contrary to our expectations, trouble getting to the facility and attending to routine tasks were not the reasons that caused respondents to eventually miss ART appointments. Most respondents reported task shifting and relying on family and their larger social networks to cover their routine responsibilities on ART clinic days. Thus, while respondents’ social support systems encountered some of the same unexpected barriers as the respondents, those same support systems were available to help respondents in accessing HIV care during routine, daily life. Further, the literature on barriers to health care in low resource settings is replete with examples of people walking long distances or struggling to pay transportation fees to access health facilities [43]. Although getting to the clinic (when not travelling away from home) was difficult for some men and women in our study, it was largely a manageable, albeit demanding, challenge—many respondents planned in advance to save money and/or routinely borrowed transportation resources from social support systems to manage the barriers of distance and travel time from home to clinic. Our research provides additional context for the common finding that wait times, distance, and transportation costs limit HIV care accessibility, and echoes research that highlights the practice of borrowing money or vehicles to visit ART clinics [15, 43].

Limited ART service availability played a large role in deterring re-engagement with HIV care among our study population. Respondents reported not being able to access ART refills because of limited ART clinic schedules, and being turned away when returning to the clinic due to staff shortages. Similarly, a recent study in Kenya documented that providers turned away clients who did not come on their scheduled appointment date, or who came in the afternoon of the same appointment day, as a way of managing high client volumes and overwhelmed health facilities [44]. Our findings reflect systemic issues related to human resource shortages and a lack of health care infrastructure in the region [45]. Integrating HIV care with other services and increasing flexibility for obtaining ART throughout the week may be essential for facilitating re-engagement in this setting.

Based on previous evidence [2, 19, 46], we expected that many respondents would feel reluctant to return to HIV care due to fear of negative responses from ART providers. Surprisingly, there was little to no indication that uncomfortable client-provider interactions were a deterrent to returning to HIV care. While respondents described both positive and negative experiences with their providers overall, they generally felt comfortable explaining to the provider why they missed their ART appointment, and did not see negative interactions as a reason to delay re-engagement with care. It is possible that our sample of successful re-engagers were uniquely resilient to negative client-provider interactions, allowing them to successfully return to care even if providers were less than welcoming.

For both men and women, their primary motivation to return to care was a desire to preserve their health and support their loved ones. However, in many cases, respondents did not make the decision to return to care alone. Consistent with other literature in the region, our findings highlight the importance of social support for HIV care engagement [15, 47, 48]. Most respondents reported that they disclosed to a friend, relative, or community member that they had missed their ART appointment and were concerned about running out of medication. As a result of disclosing, respondents’ social support networks encouraged them to return to care as soon as possible, which motivated them to re-engage.

Community health workers also played a key role in re-engagement for some respondents– a finding that is not surprising given that these health workers are central to a variety of health service utilization strategies in low resource settings [49]. Our findings underscore the subtle power that a quick visit, reminder, or a kind request from a health care worker can have. This form of outreach may be particularly important for re-engaging HIV clients who are ambivalent about their HIV care and treatment, and who could benefit from such extrinsic motivation. Community outreach and re-engagement programs should target clients with a recently missed appointment, rather than waiting for a more severe default scenario [14]—a strategy that may be considerably less intensive and more effective. Such early re-engagement efforts may be particularly important given the evidence that the longer an individual is absent from HIV care, the less invested they may become in their care and treatment [2, 9]. Finally, it was somewhat surprising that more men than women in our sample reported receiving outreach from a community health worker, although our sample is small and this finding may not be generalizable. We recommend that future research assess the role of gender in community outreach interventions.

HIV clients who miss ART appointments are often considered deviant or non-compliant by health care workers, and by the larger health system [50]. In part, this is because clients’ unsuccessful efforts to return to care are not reflected in any medical record (but were captured in our study), which limits health care workers’ perspectives of client dedication to HIV care. We found that clients’ undocumented efforts to return to care were extensive and reflect a desire to comply with the demands of HIV care and be ‘good patients’ within the health system. There is growing demand in the region for “welcome back” programs, wherein providers adopt a welcoming and non-judgmental attitude towards returning clients [7, 52]. Additional strategies are needed to increase providers’ awareness and active recognition of clients’ efforts to stay in care, which may be a simple and effective way to mitigate the shame and fear PLWH experience when returning to ART clinics [2, 22, 51]. These interventions may also promote client-provider dialogue about the client’s unique adherence challenges, and empower clients to act as a partner in achieving their HIV treatment goals [21, 50, 53].

Our findings highlight the importance of client-centered care and more flexible ART appointment schedules that acknowledge and address the challenges of competing and unexpected life events. Missed ART appointments may reflect logical rationales and calculated tradeoffs, rather than a disregard for the importance of routine HIV care [54]. Effective HIV care retention and re-engagement interventions (e.g., treatment supporters, patient navigation, and social support services) in other settings are those that consider and address the myriad factors in the lives of PLWH, outside of the clinic, that may conflict with or take higher short-term priority than attending ART appointments [55–58]. Further, self-management programs support PLWH to develop strategies to manage their treatment alongside their other life demands—an intervention that may be especially effective at retaining PLWH who experience the greatest barriers to care [49, 55, 59, 60]. Multi-month scripting for ART (3- or 6-months) and new technology such as long-acting injectable ART would also reduce the frequency of ART visits and therefore minimize competing priorities between HIV care and life outside of the clinic [61–63].

This study has several limitations that should be considered. First, we only enrolled clients who successfully re-engaged with HIV care within 60 days of a missed appointment. Therefore, our findings do not necessarily represent the barriers experienced by those who do not return to care, or who are disengaged for longer periods. Second, in conducting in-depth interviews at the ART clinic respondents may have felt pressured to express more positive sentiments about their HIV care and/or greater dedication to treatment adherence. However, we made every effort to communicate to respondents that research assistants were independent from the clinic, that their answers would not be shared with local health care workers, and responses would have no impact on their HIV care. We also included questions reframed as hypothetical examples, and included prompts that normalized a range of experiences. Third, our sample included HIV clients who recently initiated ART (< 12 months ago) and our findings do not necessarily reflect barriers and facilitators experienced by long-term ART clients. Finally, our findings are directly applicable to the context of Malawi, and may not be generalizable to other settings.

## Conclusions

Unexpected travel or caregiving led HIV-positive individuals in Malawi to miss ART appointments, and inflexible HIV care programs made it challenging for clients to re-engage in care in a timely manner. Despite these barriers, ART clients expressed a strong dedication to re-engaging with HIV care and adhering to ART in the long-term. Clients’ concerns for their health and their families, combined with different forms of social support, facilitated their timely return to HIV care. Client-centered care and flexible ART appointment schedules that accommodate unexpected life events may be critical to re-engagement and long-term retention in care.

## Data Availability

Given the depth of information contained in the interviews for this study, the data have not been made publicly available to protect the confidentiality of respondents. The interview data that support the findings of this study are available from the corresponding author upon reasonable request.

## Code Availability

The codebook, analytic memos, and the analytic codes attached to interview quotations in Atlas.ti are available from the corresponding author upon reasonable request.
